# Isolation and Characterization Identification of Edophytic Nitrogen-Fixing Bacteria from Peanut Nodules

**DOI:** 10.1155/2024/8973718

**Published:** 2024-08-31

**Authors:** Nguyen Van Chuong, Tran Le Kim Tri

**Affiliations:** ^1^ Agricultural Faculty of An Giang University, An Giang ProvinceVNU, HCM City, Vietnam; ^2^ Center Laboratory of An Giang University, An Giang ProvinceVNU, HCM City, Vietnam

## Abstract

This work was carried out to isolate and perform molecular identification and selection of endophytic nitrogen-fixing bacteria (ENFB) to be utilized as biofertilizer. In this research, nodulous samples of peanuts were collected from inside dyke areas, namely, Phuoc Hung of An Phu, An Giang, Vietnam. Ten colonies were isolated from nutrient agar plates containing YMA's medium. All isolates were rod shaped, Gram negative, and no spore creation. Biochemical tests indicated that they were obligate aerobes, catalase, oxidase, urea hydrolysis, well motile ability, and no nitrate reduction. The salt tolerance observed that most survived at 0.5% and 2% salinity (except *Enterobacter cloacae* subsp. *dissolvens* strain LMG 2683), while at 4%, only 3 isolates (*Bacillus aryabhattai* strain CM44*, Enterobacter asburiae* strain IIWM-JS-07L, and *Bacillus songklensis* strain KCa6) and at 5% only, 2 isolates survived, namely, *Enterobacter asburiae* strain IIWM-JS-07L and *Bacillus songklensis* strain KCa6. The result showed that most of ten ENFB strains could adapt to the range of 25°C and 45°C (except *Enterobacter cloacae* subsp. *dissolvens* strain LMG 2683 and Enterobacter mori strain cjy13 at 25°C). Out of ten isolates, three were finally selected for the next studies, which potentially have N-fixing ability and are utilized as biofertilizer in agricultural cultivation.

## 1. Introduction

The agricultural soil plays an important role in global food production; as a result, crop soils are intensively farmed and planted to maximize yields. Farmers use high inorganic fertilizers in large quantities for many continuous years, which are really hazardous to the health of the crop soil and the quality of agricultural products [1]. ENFB species in peanut root nodules have been studied in order to understand the symbiotic relationship for nitrogen fixation and mutual benefits [2, 3]. Endophytic nitrogen-fixing bacteria play a crucial role in crop growth by promoting plant biomass and yield, helping to meet the growing global demand for food as the population increases [4, 5]. The root nodules of legumes are formed through a symbiotic relationship with ENFB called as rhizobia. These nodules have specialized structures and functions that facilitate the conversion of atmospheric N_2_ into plant-usable forms such as NH_4_^+^ and NO_3_^−^ by the ENFB strains through a process called as biological nitrogen fixation. The ENFB utilization for raising the crop yield is an economic and sustainable method, which reduce the utilization of inorganic fertilizers and pesticides [6]. Endophytic nitrogen-fixing bacteria are popularly used as natural nitrogen fertilizers, promoting sustainable agricultural practices [7]. Many prior research studies have proven that the peanut plant associated with ENFB strains could improve farmland fertility by fixing air nitrogen [8, 9]. Peanuts are able to take the nitrogen of air by the symbiotic relationship with ENFB, which resides inside peanut roots' nodules [10]. Symbiotic N-fixation between rhizobia and legumes plays a crucial role in the ecological interaction between soil and crops, and it determines nutrient composition, biodiversity, and the succession of subsequent generations. They annually contribute around 100–290 million N tons to farmlands and crops. This natural N resource supports natural ecosystems, enhances plant growth, and holds global agronomic importance. In addition, the bacteria have increased the diversity of legumes due to this nitrogen fixation ability [8, 10]. Particular significance for the interaction between plants and biology is the legume-rhizobia reciprocity, which fix N_2_ sources of air to reclaim carbon supplied by legumes. This benefit interaction ensures regularly accurate nutrient cycles in natural ecosystems by supporting nitrogen in an agricultural cultivation [11]. The increasing cost of using various types of chemical fertilizers and the negative impacts of pesticides have led to reduced profits and quality of agricultural products. This problem requires a solution through the use of organic products and natural plant protection methods. The use of plant growth-promoting bacteria to make plants healthier and improve soil quality has become an attractive method for developing sustainable farming systems due to their unique properties for the environment, production, low cost, and reduced consumption of nonrenewable fertilizer energy [12, 13]. From the abovementioned reasons, this study first isolated and characterized native ENFB from peanut nodules. These selected strains could become a base product to improve crop productivity and raise soil fertility.

## 2. Materials and Methods

### 2.1. Nodule Collection

Nodule samples of peanuts were from peanuts plants in Phuoc Hung commune, An Phu district, An Giang Province. Nodules, which were taken from peanut roots, were cut from the tumor using scissors and then the surface of the nodules were disinfected with CaOCl_2_ solution (4%) for 180 seconds before being cleaned with fresh water for 10 seconds. The collected nodule samples were in the size of 02 –03 mm in diameter. The light or pink nodules indicated that a community of nitrogen-fixing bacteria were acting and living inside [14, 15]. Later, nodules were soaked in 70% Alc. solution for 3 minutes and again washed with sterile distilled water. The clean nodules placed into an Eppendorf, containing sterile distilled water to form suspension. The above suspension was diluted from 10^−2^ to 10^−6^ and inoculated into 3 Petri dishes of YMA medium. The plates after inoculation were incubated at room temperature for 4-5 days, followed by observation for the growth of colonies, recording for the results and inoculation and purification [16].

### 2.2. ENFB Isolation

The nutrient medium composition of YMA consisted of yeast extract, mannitol, K_2_HPO_4_, MgSO_4_, NaCl, agar, pH = 7, and adding Congo red solution to obtain the 25 mg·L^−1^ content. YMA medium plates containing ENFB colonies were incubated at 28°C for 4 days. It was continued by streaking the separated isolates until forming completely pure colonies from white to pink and malleable and shimmery. The colonies of ENFB were then extracted from most other soil bacteria by filtration through YMA [14, 15].

The isolated ENFB genus was further classified through a number of selective media since most of the ENFB strains were Gram-negative and rod-shaped bacteria.

- YMA-BTB medium: this can identify ENFB colonies fast because of turning agar to slowly blue color
- GPA-BCP medium: in this medium, ENFB strains poorly grew on because of high pH, used for checking the isolate purity [17].
- Lactose agar medium: this medium was to check the yellow ring around the colonies after being flooded with reagent of Benedict, confirming the ENFB presence because of converting lactose to 3-ketolactose. It was called as the keta-lactose test [18].
- Hofer's alkaline medium: this medium was used for ENFB strains isolation, which could grow at high pH such pH > 6.5 [19].
- N-free medium: this medium was used by isolating free-living ENFB strains, which could utilize air N to form protein synthesis. In this research, it was used for testing colonies, which could develop N-free medium or not [20]. Checking Biochemistry was conducted using the ASM technology [21].

**Figure 1:**
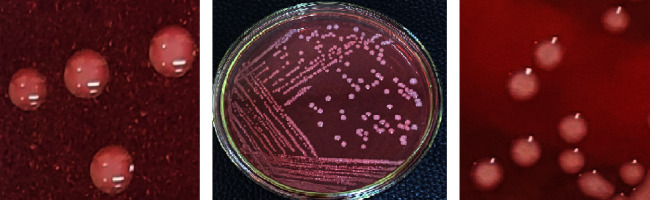
Colony morphology of *Bacillus aryabhattai* strain CM44 (a)*, Enterobacter asburiae* strain IIWM-JS-07L (b), and *Bacillus songklensis* strain KCa6 (c).

As shown in Figure 1, three pure colonies for endophytic bacterial genera were isolated from peanut nodules, which were selected as potential endophytic bacterial isolates with distinct morphological and colony color. The isolation and genomic identification of endophytic bacterial genera from peanut nodules by the YMA nutrient medium was conducted for the identification of isolates and Gram symbols for identification of their biological properties.

### 2.3. 16S rDNA Sequencing

Pure ENFB colonies were placed in Eppendorf tubes for DNA extraction, which were used by the GeneJET Genomic DNA Purification Kits from Thermal Scientific™. The 16S rDNA genes of the isolates was amplified by PCR using the primer set 20F 5′-CTACGGCAAGGCGACGCTGACG-3′ and 1500R 5′- GGTTACCTTGTTACGACTT-3′ [16, 22]. The sequence data were analyzed using MEGA software and compared to the most similar sequence in GenBank using the BLAST technique with an identity threshold of greater than 99.70% [23].

### 2.4. ENFB Characterization

The ENFB species were purified and characterized by Gram staining, catalase and oxidase tests, and macroscopic and microscopic observations. Their growth on YMA containing bromothymol blue was observed as well. In addition, the isolated species were tested for their resistance to heat and salinity, which are main factors for screening ENFB for the introducing an aim to them into degraded soils.

### 2.5. Thermal Tolerance

To determine the optimal incubation temperature, a set of 12 tubes containing YMA medium were prepared. Four tubes were prepared for each temperature and incubated at 25, 37, and 45 ± 1°C in an incubator. The growth of bacterial colonies was daily monitored for 7 days. The thermos tolerance test of the isolated ENFB species was performed, which were streaked on YMA agar plates and incubated at temperature ranging from 25, 37 and 45°C. The experiments were performed in quadruplicate for each species and the growth of colonies was recorded for one week [24].

### 2.6. Saline Tolerance

Nutrient agar containing different the NaCl concentrations (0.5%, 2%, 3%, 4%, and 5%) were inoculated by ten isolated colonies and incubated at 28°C with four replications and recorded the results after a week [24].

## 3. Results

### 3.1. Isolation and Characterization Identification

Nodulous samples were collected from Phuoc Hung commune, An Phu district, An Giang, Vietnam. These collected nodules were isolated by the new technologies for ENFB [25]. The isolation and genomic identification of rhizosphere N-fixing bacteria from peanut nodules by the YMA nutrient medium was conducted for the identification of isolates and Gram symbols for identification of their biological properties.  Table 1 presents the identified species, noting that all isolates were negative for Gram staining after flooding.

The sequencing results (Table 2) showed that the similarity of the ten selected species was ≥99.5% compared to the standard ENFB species. The NCBI database (https://www.ncbi.nlm.nih.gov/genbank/) was used to identify their highest similarity.

Table 3 illustrates the adaptability of ten ENFB strains to the temperature range of 25–45°C, within which they were capable of surviving and growing. Notably, the optimal temperature range for their development fell between 37 and 45°C. Excessive or insufficient temperatures in agricultural environments can adversely affect plant growth and nodule formation, ultimately compromising the nitrogen-fixing efficiency of ENFB [26]. YMA medium containing different NaCl concentrations (0.5%, 2%, 3%, 4%, and 5%) were inoculated at 28°C for 48 hours. The isolates grew in the different NaCl concentrations, which were then observed and compared together (from low to high NaCl concentrations). The salt tolerance test observed that most survived at 0.5% and 2% salinity (except *Enterobacter cloacae* subsp. *dissolvens* strain LMG 2683 and *Enterobacter mori* strain cjy13), while at 4%, only 3 isolates (species: B2B, V1 and 18) and at 5%, only 2 isolates survived, namely, V1 and 18. Most of ten ENFB strains could adapt to the range of 25°C and 45°C (except *Enterobacter cloacae* subsp*. dissolvens* strain LMG 2683 *and Enterobacter mori* strain cjy13 at 25°C) [27]. In this study, the pH effect of selected colonies on the YEMA semisolid medium (acidified with H_2_SO_4_) was investigated after 5 days of incubating. The results showed that seven out of ten selected strains exhibited weak growth at pH 4.5 (except for B2B, V1, and 18). Ten selected species grew well at pH = 7. However, the colonies of B2B, V1, V2, and 18 were able to survive on pH 8.5 (Table 3).

**Figure 2:**
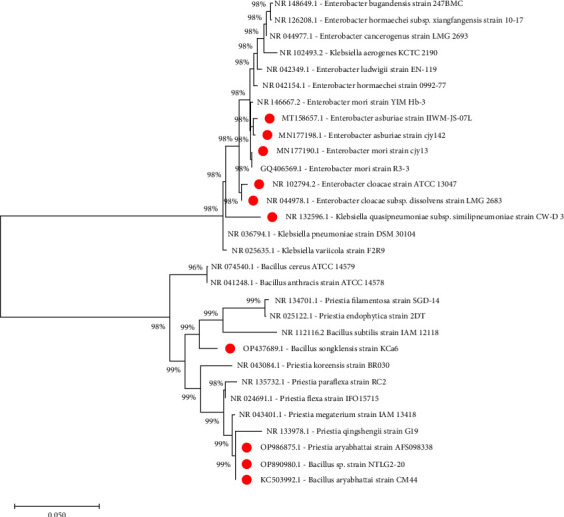
Phylogenetic tree of partial 16S rRNA sequences of ENFB strains isolated from peanut nodules along with the sequences from selected references strains. The tree was constructed by the neighbor-joining method using MEGA 11. The scale bar corresponded to 0.050 substitutions per nucleotide position. Numbers on the branches were bootstrap percentages. GenBank accession numbers are shown above.

Figure 2 shows that the three selected species, including *Bacillus aryabhattai* strain CM44, *Enterobacter asburiae* strain IIWM-JS-07L, and *Bacillus songklensis* strain KCa6, exhibited remarkable characteristics when being tested for their ability to withstand heat, salinity, and other conditions. Furthermore, they shared a high degree of similarity with high nitrogen-fixing species. These species were selected based on previous screenings and were represented in the phylogenetic tree. The phylogenetic determination used three selected isolates with reference neighboring sequences from the previous database. A close relationship of these branches was closely grouped together with the genera of *Bacillus aryabhattai* strain CM44, *Enterobacter asburiae* strain IIWM-JS-07L, and *Bacillus songklensis* strain KCa6.

## 4. Discussion

On Hofer medium, which had a high pH, the ability of selected species to grow well was considered as a key factor in assessing its potential to belong to the group of ENFB species [28]. All ten obtained strains with clear pink colonies on YMA. It is a main characteristic of *Rhizobium* strain, already explained by Somasegaran and Hoben [15]. The test of GPA-BCP medium aimed to check a culture purity of a highly nutritious medium. The results, as shown in Table 1, showed that all ENFB isolates were significantly developed on this medium. On contrary, other genus were weakly developed [29]. All identified ENFB species were checked for the nitrogen-fixing ability by Burk medium (without N) to determine their genera size. This result represented that isolated species developed well on agar medium (without nitrogen). These ENFB species were the highest priority selection to study on the field experiments [30–32]. The tests of oxidase and catalase showed that all rhizobial strains had positive results. These results were consistent with previous studies revealing that rhizobial strains usually give positive results for the oxidase and catalase reaction [33, 34]. The test of chemical traits showed different results dependent on each rhizobial species (Table 4). However, theses result proved that different factors of ENFB species were highly affected by their living environment [35, 36]. The 16S rRNA sequences of the ten selected strains, as presented in Table 2, were derived from the rhizosphere of peanut plants. This analysis, along with other cutting-edge molecular techniques, has facilitated the discovery of numerous new ENFB species. These findings have significantly expanded our knowledge of ENFB diversity and their valuable attributes. Overall, learning these new techniques is a means to enhance the reliability and effectiveness of molecular research and applications. [37]. The selected ENFB species could have the high adaption of NaCl concentration up to 3-4% [24] or even to 5% (w/v) NaCl [38]. However, NaCl concentration was below 1% (w/v), which was suitable for most ENFB species. Ten obtained species were suitable in pH from 5 to 8 [37, 39].

## 5. Conclusions

Ten ENFB species were isolated, identified, and tested by various biochemical and molecular assays. Three selected potential species consisted of *Bacillus aryabhattai* strain CM44, *Enterobacter asburiae* strain IIWM-JS-07L, and *Bacillus songklensis* strain KCa6, which were tested for the abilities of thermal, saline pH tolerance, and for their categorization into groups to be used for further field studies to the ultimate goal of developing inoculants in future. Understanding ENFB and their host plant origins has enabled us to make more extensive and confident use of these positive species, as long as their optimal conditions are still well concerned.

## Figures and Tables

**Table 1:** Identification of ENFB from peanut nodules.

Symbol	Identity (rod shape)	YMA (clear pink)	YMA-BTB (yellow)	GPA	Hofer agar	Burk agar	Genus
B2B	(−) gram	(+)	(+)	(+)	(−)	(++)	ENFB
V1	(−) gram	(+)	(+)	(+)	(−)	(++)	ENFB
V2	(−) gram	(+)	(+)	(+)	(−)	(++)	ENFB
18	(−) gram	(+)	(+)	(+)	(−)	(++)	ENFB
V4	(−) gram	(+)	(+)	(+)	(−)	(+)	ENFB
V5	(−) gram	(+)	(+)	(+)	(−)	(++)	ENFB
2	(−) gram	(+)	(+)	(+)	(−)	(+)	ENFB
3	(−) gram	(+)	(+)	(+)	(−)	(+)	ENFB
4	(−) gram	(+)	(+)	(+)	(−)	(+)	ENFB
5	(−) gram	(+)	(+)	(+)	(−)	(+)	ENFB

Note: (−): negative gram; (−): no reaction; (++): strong reaction.

**Table 2:** Identification of ENFB isolates by partial sequencing of 16S rRNA.

Symbol	Strains	Similarity (%)	16s rRNA sequence (5′ ⟶ 3′)	GenBank accession no.
B2B	*Bacillus aryabhattai* strain CM44	100	1–1457 bp	KC503992.1
V1	*Enterobacter asburiae* strain IIWM-JS-07L	99.74	1–1406 bp	MT158657.1
V2	*Klebsiella quasipneumoniae* subsp. *similipneumoniae* strain CW-D 3	99.65	1–1456 bp	NR_132596.1
18	*Bacillus songklensis* strain KCa6	99.93	1–1495 bp	OP437689.1
V4	*Enterobacter cloacae* subsp*. dissolvens* strain LMG 2683	99.65	1–1543 bp	NR_044978.1
V5	*Enterobacter cloacae* strain ATCC 13047	99.65	1–1543 bp	NR_102794.2
2	*Bacillus* sp. strain NTLG2-20	100	1–1424 bp	OP890980.1
3	*Priestia aryabhattai* strain AFS098338	99.92	1–1528 bp	OP986875.1
4	*Enterobacter asburiae* strain cjy142	99.63	1–1380 bp	MN177198.1
5	*Enterobacter mori* strain cjy13	99.93	1–1442 bp	MN177190.1

**Table 3:** Tolerance of selected strains to high temperature, salt, and pH in vitro [27].

Symbol	References species	NaCl (%)					Temperature (°C)			pH		
		0.5	2	3	4	5	25	37	45	4.5	7	8.5
B2B	*Bacillus aryabhattai* strain CM44	++	+	++	+	−	++	++	+	++	++	+
V1	*Enterobacter asburiae* strain IIWM-JS-07L	++	++	++	++	+	++	++	+	++	++	++
V2	*Klebsiella quasipneumoniae* subsp. *similipneumoniae* strain CW-D 3	++	+	+	−	−	++	++	+	+	++	++
18	*Bacillus songklensis* strain KCa6	++	+	++	+	+	++	++	+	++	++	+
V4	*Enterobacter cloacae* subsp*. dissolvens* strain LMG 2683	++	−	−	−	−	−	++	+	+	++	−
V5	*Enterobacter cloacae* strain ATCC 13047	++	+	−	−	−	++	++	+	+	++	−
2	*Bacillus* sp. strain NTLG2-20	++	+	+	−	−	++	++	+	+	++	−
3	*Priestia aryabhattai* strain AFS098338	++	+	−	−	−	+	++	+	+	++	−
4	*Enterobacter asburiae* strain cjy142	++	+	−	−	−	++	++	+	+	++	−
5	*Enterobacter mori* strain cjy13	++	−	−	−	−	−	++	+	+	++	−

**Table 4:** Biochemical tests of ENFB.

Symbol	Oxidase	Catalase	Urea hydrolysis	Nitrate reduction	Citrate utilization
B2B	(+)	(+)	(+)	(−)	(+)
V1	(+)	(+)	(+)	(−)	(+)
V2	(+)	(+)	(+)	(−)	(+)
18	(+)	(+)	(+)	(−)	(+)
V4	(+)	(+)	(+)	(−)	(+)
V5	(+)	(+)	(+)	(−)	(+)
2	(+)	(+)	(+)	(−)	(+)
3	(+)	(+)	(+)	(−)	(+)
4	(+)	(+)	(+)	(−)	(+)
5	(+)	(+)	(+)	(−)	(−)

Note: (−): no reaction; (+): reaction.

## Data Availability

The data used to support the findings of this study are available from the corresponding author upon reasonable request.

## Supplementary Materials

Supplementary Materials The sequence data of ten endophytic bacteria were analyzed using 16S ribosome RNA gene partial sequecen, which was used by MEGA software and then compared to base on the most similar sequence found in GenBank using the BLAST technique with an identity threshold of greater than 99.70%.
